# The Global Hidden Hunger Indices and Maps: An Advocacy Tool for Action

**DOI:** 10.1371/journal.pone.0067860

**Published:** 2013-06-12

**Authors:** Sumithra Muthayya, Jee Hyun Rah, Jonathan D. Sugimoto, Franz F. Roos, Klaus Kraemer, Robert E. Black

**Affiliations:** 1 Sax Institute, Sydney, Australia; 2 United Nations Children’s Fund (UNICEF) India, New Delhi, India; 3 Department of Epidemiology, College of Public Health and Health Professions and the College of Medicine, University of Florida, Gainesville, Florida, United States of America; 4 Vaccine and Infectious Disease Division, Fred Hutchinson Cancer Research Center, Seattle, Washington, United States of America; 5 DSM Nutritional Products Ltd, Kaiseraugst, Switzerland; 6 *Sight and Life*, Kaiseraugst, Switzerland; 7 Department of International Health, Johns Hopkins Bloomberg School of Public Health, Baltimore, Maryland, United States of America; Kenya Medical Research Institute - Wellcome Trust Research Programme, Kenya

## Abstract

The unified global efforts to mitigate the high burden of vitamin and mineral deficiency, known as hidden hunger, in populations around the world are crucial to the achievement of most of the Millennium Development Goals (MDGs). We developed indices and maps of global hidden hunger to help prioritize program assistance, and to serve as an evidence-based global advocacy tool. Two types of hidden hunger indices and maps were created based on i) national prevalence data on stunting, anemia due to iron deficiency, and low serum retinol levels among preschool-aged children in 149 countries; and ii) estimates of Disability Adjusted Life Years (DALYs) attributed to micronutrient deficiencies in 136 countries. A number of countries in sub-Saharan Africa, as well as India and Afghanistan, had an alarmingly high level of hidden hunger, with stunting, iron deficiency anemia, and vitamin A deficiency all being highly prevalent. The total DALY rates per 100,000 population, attributed to micronutrient deficiencies, were generally the highest in sub-Saharan African countries. In 36 countries, home to 90% of the world’s stunted children, deficiencies of micronutrients were responsible for 1.5-12% of the total DALYs. The pattern and magnitude of iodine deficiency did not conform to that of other micronutrients. The greatest proportions of children with iodine deficiency were in the Eastern Mediterranean (46.6%), European (44.2%), and African (40.4%) regions. The current indices and maps provide crucial data to optimize the prioritization of program assistance addressing global multiple micronutrient deficiencies. Moreover, the indices and maps serve as a useful advocacy tool in the call for increased commitments to scale up effective nutrition interventions.

## Introduction

Globally, an estimated two billion lives are affected by a chronic deficiency of essential vitamins and minerals (micronutrients), collectively known as hidden hunger [1–4]. As the term hidden hunger indicates, the signs of undernutrition and hunger are less overtly visible in those affected by it. Nevertheless, its negative and often lifelong consequences for health, productivity, and mental development are devastating [5]. Young children and women of reproductive age living in low-income countries are the most vulnerable. In recent years, volatile food prices and climate change have led to changes in dietary intake, with a shift away from foods which are rich in micronutrients, while retaining the consumption of low-micronutrient-containing staple foods which are relatively less expensive [6,7]. Consequently, an increasing proportion of the world’s population may be at risk of hidden hunger, with potential significant negative consequences for both global health and economic growth.

Worldwide, the most widespread micronutrient deficiencies are of iron, zinc, vitamin A, iodine, and folate, but deficiencies of vitamin B12 and other B vitamins also commonly occur. In developing countries, multiple micronutrient deficiencies often occur together in the same population [8]. These deficiencies account for approximately 7% of the global disease burden annually [9]. The 2008 *Lancet* series on Maternal and Child Undernutrition reported that deficiencies of vitamin A and zinc were responsible for 0.6 million and 0.4 million child deaths respectively, and a combined 9% of global childhood Disability Adjusted Life Years (DALYs). Iron deficiency alone was associated with 115,000 maternal deaths [9]. Iron and iodine deficiencies were related to cognitive impairment, but resulted in few child deaths. Even mild to moderate deficiencies of micronutrients lead to impaired intellectual and psychomotor development, poor physical growth, increased morbidity from infectious diseases in infants and young children, and decreased work productivity in adulthood [10–18].

Global databases on anemia and iron, vitamin A and iodine deficiency have provided useful information on the magnitude and distribution of individual deficiencies [2–4]. However, a strong evidence base of the burden of collective micronutrient deficiency and its contributions to disease, both nationally and globally, is lacking. Such information will enable the development of appropriate interventions, which would effectively target those populations most affected by multiple micronutrient deficiencies. Interventions targeted at alleviating individual micronutrient deficiencies have achieved mixed results in their effectiveness [19–21]. A sustainable strategy that tackles co-existing deficiencies, such as home fortification with micronutrient powders for preschool-age children and staple food fortification for the general population, is therefore urgently required. Evidence has suggested that fortification with multiple micronutrients could be one of the most sustainable and cost-effective development investments [22].

Indices and maps are useful tools for public health advocacy and planning, and can guide policy decisions. This paper describes the development of global indices and maps depicting hidden hunger, reflecting both the prevalence of multiple micronutrient deficiencies and the associated disease burden, to serve as a tool to stimulate global efforts towards scaling up nutrition interventions. By highlighting the global hidden hunger hotspots and providing a ranking index of affected countries, the maps are expected to be useful in informing strategies for unified efforts to eliminate hidden hunger. It is anticipated that these indices and maps will enable public health scientists and policy makers to prioritize program assistance for those countries most affected by hidden hunger.

## Materials and Methods

Two separate datasets were compiled for the development of hidden hunger indices and maps: i) a database of the most up-to-date national prevalence estimates of anemia, stunting, vitamin A deficiency (VAD) in pre-school aged children, and iodine deficiency (ID) in school-aged children, for 190 countries for the years 1999-2009; and ii) data of the recent DALY estimates attributed to deficiencies of iron, zinc, vitamin A, and iodine for 192 countries. Using these datasets, hidden hunger maps and indices were created by i) combining national prevalence estimates of anemia, stunting, and VAD for preschool-age, children, together with separately added estimates of ID for school-age children; and ii) combining country-wide DALY estimates attributed to deficiencies of iron, zinc, and vitamin A for the population.

Data on stunting, anemia, VAD, and ID prevalence were chosen on the basis of their contribution to hidden hunger, as well as the global availability of nationally-representative estimates. Deficiencies of folate and vitamin B12 were excluded from the dataset, due to the limited availability of national data.

In the absence of national data on iron deficiency or iron deficiency anemia (IDA), prevalence estimates for anemia were used, recognizing that anemia could reflect both nutritional deficiencies and non-nutritional factors, such as infections, inflammation, and thalassemia or hemoglobinopathy. In this analysis, it was assumed that 60% of anemia was due to iron deficiency in non-malaria settings and 50% in malaria endemic areas [23,24]. Stunting prevalence was used as a proxy of zinc deficiency, as recommended by the International Zinc Nutrition Consultative Group [25].

Estimates of anemia prevalence were obtained from two main sources: i) the World Health Organization (WHO) Global Database on Anemia [2], a part of the Vitamin and Mineral Nutrition Information System (VMNIS); and ii) Demographic and Health Surveys (DHS). Only nationally representative prevalence data for preschool-age children (0-4.99 years) were included, as this was the most vulnerable age group and anemia prevalence in children had strong correlations with the corresponding anemia rates in pregnant women (r=0.83), and in women of reproductive age (r=0.82). Wherever possible, data on children below 0.5 years were excluded, since the cut-off for anemia is not defined in this age group. As an exception, the national rural data for Bangladesh and data aggregated from several state surveys for Brazil were included as national estimates. For countries without national survey data, regression-based estimates developed by the WHO were used in our analyses [2].

Data on VAD were obtained from the WHO Global Database on VAD, part of the VMNIS [4]. Only national prevalences of low serum (or plasma) retinol concentration, using a cut-off of <0.70 µmol/L, were used. Prevalence estimates for preschool-age children were used because national survey data for other population groups, such as pregnant women, were limited. For countries lacking national survey data, regression-based estimates developed by the WHO were used in our analyses [4]. All countries (n=37) with a 2005 gross domestic product (GDP) ≥US$ 15,000 were assumed to be free from VAD, and therefore did not have serum retinol data.

Data on ID were extracted from the WHO Global Database on ID, part of the VMNIS, and from the Demographic and Health Surveys (DHS) and Multiple Indicator Cluster Surveys (MICS) [3]. Additional new national data available since 2007 for 50 countries were also included [25]. The prevalence of ID was defined as the proportion of school-age children having a urinary iodine (UI) concentration <100 µg/L. For countries where only the median UI was reported, regression estimates of ID prevalence derived from UI concentration studies compiled in the WHO VMNIS database were used. However, exceptions were made for countries with pooled data from multiple surveys (Italy, Spain, and the Russian Federation), with national data on ID restricted to the urban population (New Zealand, Sudan), and with national data for population groups other than only school age (the Czech Republic, France, Kazakhstan, Oman, Slovenia, Tajikistan, Ukraine, and the UK) [3,26]. For countries without national survey data, ID prevalence estimates were left missing.

Nationally representative data on moderate and severe stunting among preschool-age children were extracted from the WHO database on child growth and malnutrition, the UNICEF global database on child growth, and the DHS and MICS surveys. Stunting was defined as height-for-age z-scores below -2 of the new WHO growth reference standards [27]. For the few countries with missing values (n=11), the mean stunting prevalence of countries in the same WHO region, weighted by the population size for 2009, was assigned as the best estimate.

For the DALY dataset, the most recent DALY estimates attributed to deficiencies of iron, zinc, and vitamin A in 136 countries were compiled. DALYs are the sum of Years of Life Lost (YLLs) and Years Lived with Disability (YLDs) for incident conditions. For the calculation of these DALYs, expert working groups conducted comprehensive reviews of data on risk-factor exposure and hazard for 14 epidemiological sub-regions of the world, by age and sex. Data reflected the current status of mortality, the prevalence of micronutrient deficiency, and existing micronutrient programs at the date of the calculations. The calculations also adjusted the estimates in order not to double-count. The contribution of a risk factor to disease or mortality was expressed as the fraction of disease or death attributable to the risk factor in a population, and was referred to as the population-attributable fraction (PAF). When estimating the total effects of individual distal factors on disease, both mediated and direct effects were considered because, in the presence of mediated effects, controlling for the intermediate factor would attenuate the effects of the more distal factors. When estimating the joint effects of the more distal factors and the intermediate factors, the mediated and direct effects were separated. Further details of the estimation of DALYs attributed to micronutrient deficiencies are described elsewhere [8,24,28].

In addition to micronutrient deficiency variables, national data on important proximate determinants of hidden hunger were compiled. These include the percentage of the population with inadequate dietary energy intakes estimated by the Food and Agriculture Organization (FAO), and the Human Development Index (HDI) and Multidimensional Poverty Index (MPI) of the United Nations Development Programme (UNDP) [29,30]. The HDI provides a composite measure of three basic dimensions of human development: a long and healthy life, education, and the standard of living. The MPI identifies overlapping deprivations at a household level across the same three dimensions as the HDI, and shows the average number of poor people and deprivations with which poor households contend. Countries were grouped by WHO region.

### Statistical Analysis

We defined the Hidden Hunger Index (HHI-PD) for preschool-age children as the average of three deficiency prevalence estimates: preschool children affected by stunting, anemia due to iron deficiency, and VAD. The three components were equally weighted (HHI-PD score = [stunting (%) + anemia (%) + low serum retinol (%)]/3). The iodine deficiency estimates for school-age children were not included in the HHI estimation due to its weak correlations with other micronutrient deficiencies (r=0.01-0.18). The HHI-PD score ranged between the best and worst possible scores of 0 and 100, respectively. Applying arbitrary cut-offs, HHI-PD scores between 0 and 19.9 were considered mild, 20-34.9 as moderate, 35-44.9 as severe, and 45-100 as alarmingly high. Highly developed countries with a 2007 Human Development Index (HDI) score above 0.9 (n=41) were assumed to have a low prevalence of micronutrient deficiencies, and were therefore excluded from this analysis. The 2007 Life Expectancy Index was used as a substitute for countries with missing HDI scores. In addition, a few countries with neither VAD nor anemia prevalence data nor regression estimates were excluded.

The Hidden Hunger Indices which reflected the global disease burden were computed in two different ways, as i) the total hidden-hunger-associated DALYs per 100,000 population (HHI-DBa); and ii) the total hidden-hunger-associated DALYs per country (HHI-DBu). The DALYs attributed to ID were not included in the HHI estimation due to incomplete data for several countries, its weak correlations with other micronutrient deficiencies, and its relatively small contribution to the total DALY-based HHI. The associations between HHI-PD and both HHI-DBa and indicators of human development and inadequate dietary energy intakes were examined using the Spearman rank correlation coefficient.

## Results

A total of 149 countries with a 2007 HDI value <0.9 were included in the HHI-PD estimation (Appendix **S1**). A large proportion of the 41 countries excluded (HDI≥0.9) were located in Europe, and had a national prevalence of stunting and VAD missing or, when available, had prevalence estimates of <10% for stunting and anemia due to iron deficiency (Appendix **S2**).

**Figure 1:**
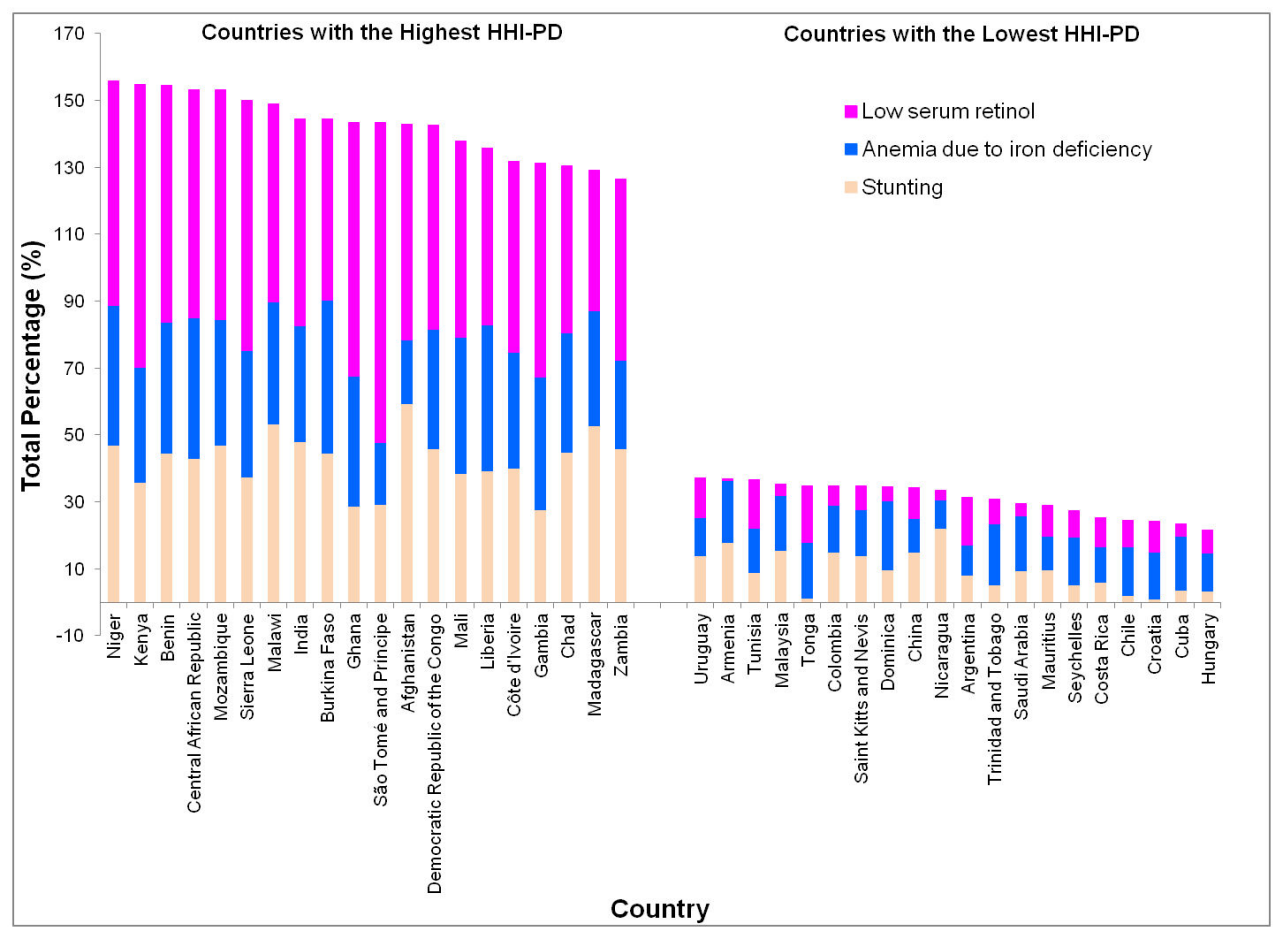
Prevalence of stunting, iron deficiency anemia, and low serum retinol in the countries with the 20 highest and lowest hidden hunger index based on the prevalence estimates (HHI-PD). The hidden hunger index (HHI-PD) was estimated based on national estimates of the prevalence of stunting, anemia due to iron deficiency, and low serum retinol concentration.

The country with the highest HHI-PD score for preschool-age children was Niger and the lowest was Hungary (Figure 1). Of the 20 countries with the highest HHI-PD scores, 18 were in sub-Saharan Africa and two, India and Afghanistan, were in Asia. The majority of these countries had child stunting, anemia due to iron deficiency, and VAD rates among preschool-age children of over 40%, 30%, and 50%, respectively (Figure 1). The majority of the countries with low HHI-PD scores had child stunting and VAD prevalence of less than 10%.

**Figure 2:**
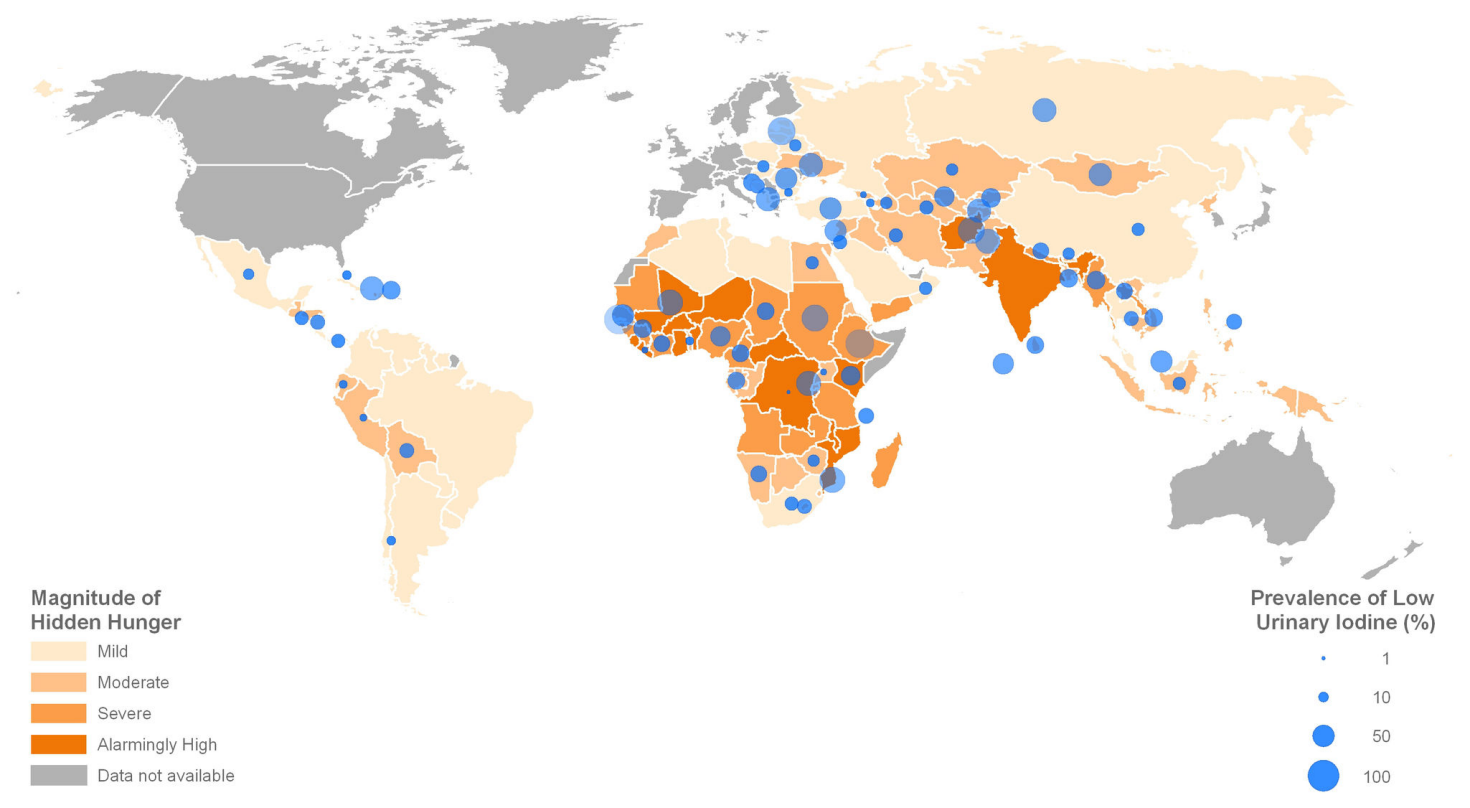
Global map presenting hidden hunger index based on the prevalence estimates (HHI-PD) in 149 countries and prevalence of low urinary iodine concentration in 90 countries with 2007 Human Development Index <0.9. The hidden hunger index HHI-PD was estimated based on national estimates of the prevalence of stunting, anemia due to iron deficiency, and low serum retinol concentration.

Globally, there were hot spots of hidden hunger, with the prevalence being alarmingly high in sub-Saharan Africa, and severe in many countries in South-Central/South-East Asia (Figure 2). Most South American countries only had a mild-to-moderate degree of hidden hunger. In many countries, ID prevalence in school-age children did not conform to the magnitude of hidden hunger. For instance, the Democratic Republic of the Congo and Liberia, both with an alarmingly high degree of hidden hunger, had low ID prevalences of 1.5% and 3.5%, respectively. In addition, in Latvia, the Russian Federation, Estonia, and Malaysia, all of which exhibited a mild degree of hidden hunger, ID prevalence was as high as 76.8, 58.6, 67.0 and 48.2%, respectively.

**Figure 3:**
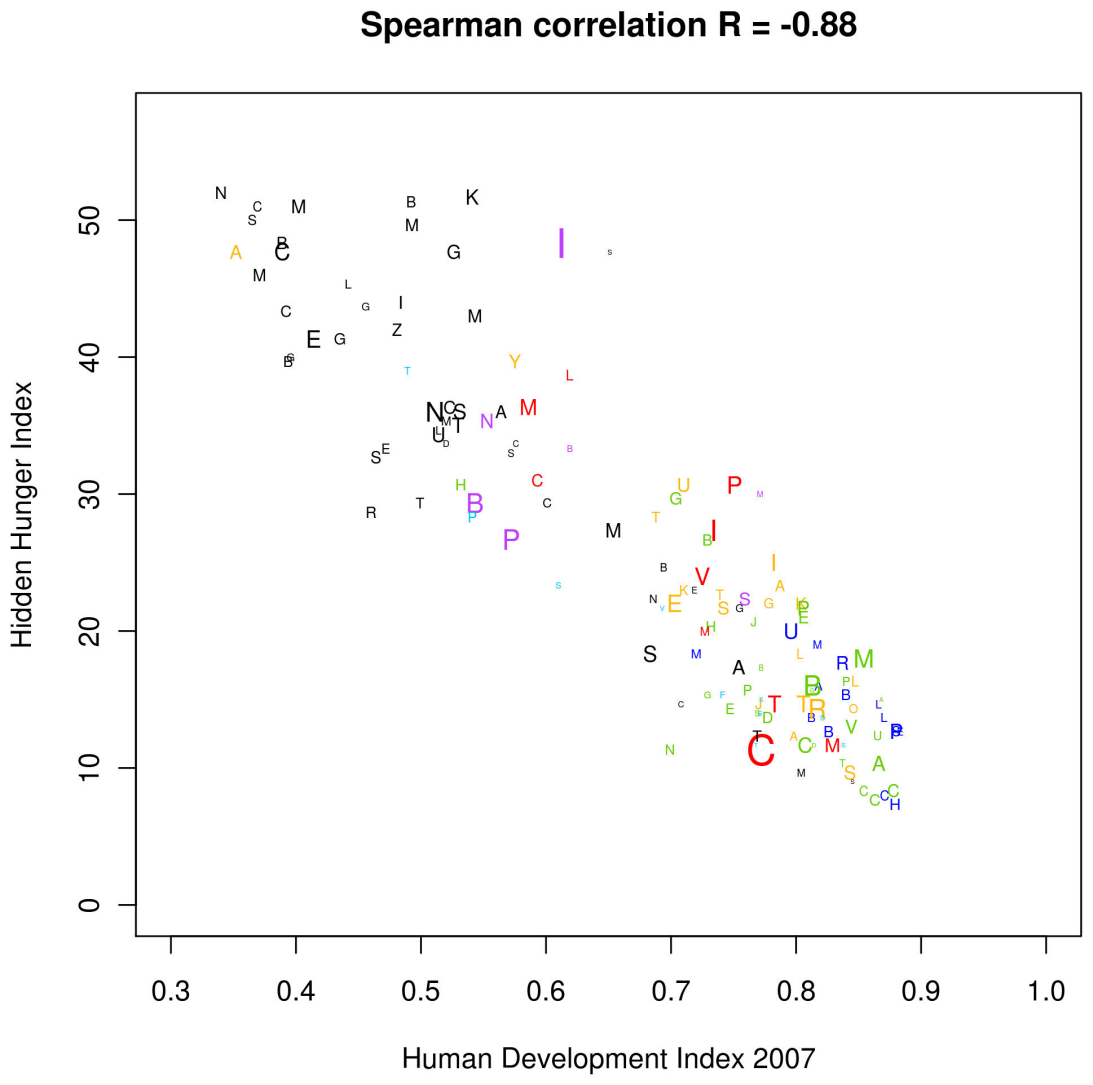
The association between hidden hunger index based on the prevalence estimates (HHI-PD) and 2007 Human Development Index (HDI). The alphabet characters symbolize the first initial of each country; the font sizes are proportional to the population size. Font color represents different regions, such that black represents Africa; red, East Asia; yellow, West Central Asia; green, Central and South America; turquoise, the Pacific Islands, and the Caribbean; purple, South Asia; and blue, Europe, North America, Australia, and New Zealand.

**Figure 4:**
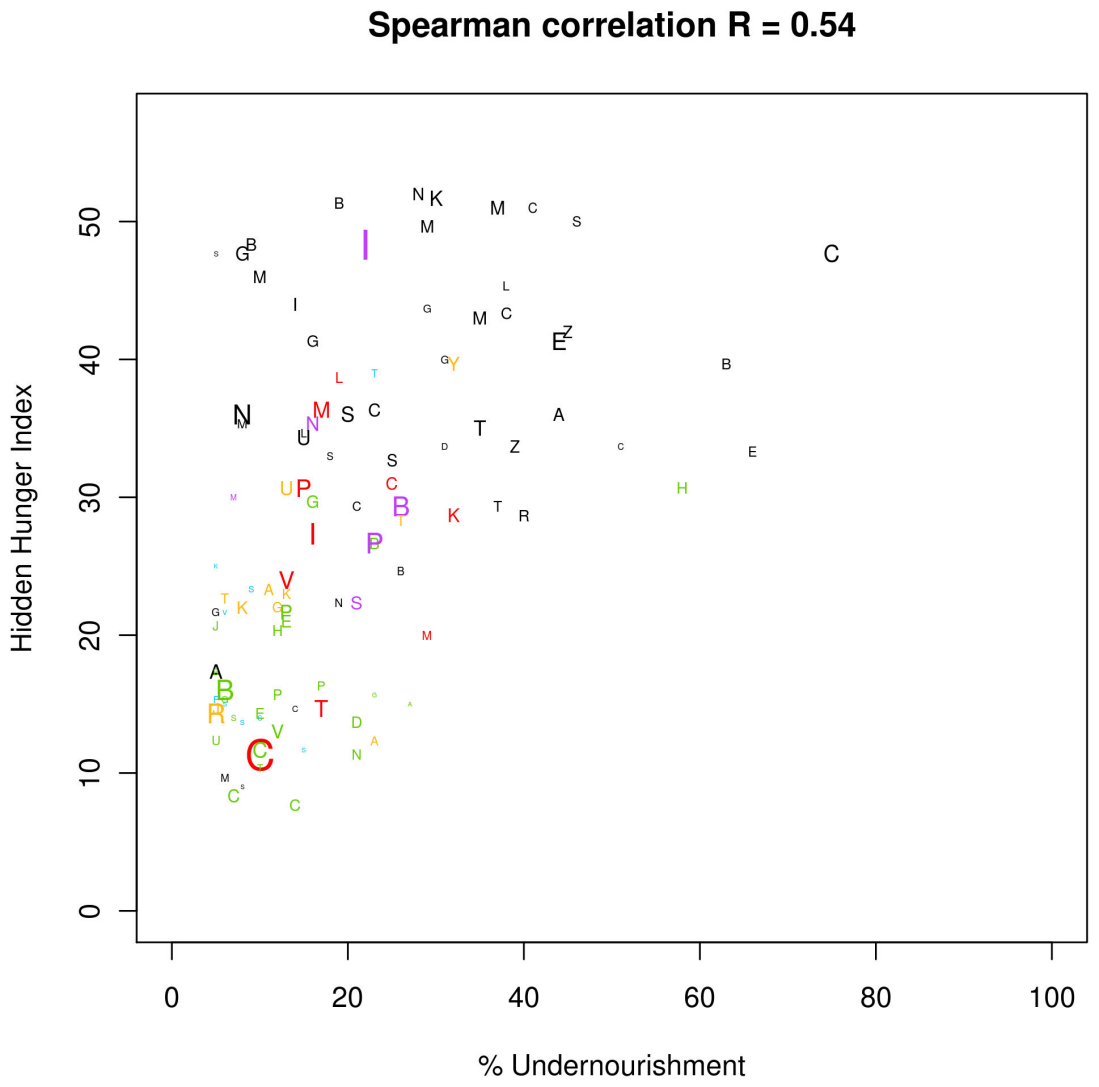
The association between hidden hunger index based on the prevalence estimates (HHI-PD) and the proportion of the population with inadequate dietary energy. The alphabet characters symbolize the first initial of each country; the font sizes are proportional to the population size. Font color represents different regions, such that black represents Africa; red, East Asia; yellow, West Central Asia; green, Central and South America; turquoise, the Pacific Islands, and the Caribbean; purple, South Asia; and blue, Europe, North America, Australia, and New Zealand.

A strong inverse association was noted between the HHI-PD and 2007 HDI values (Figure 3). There was a moderate positive association between HHI-PD and the proportion of the population with inadequate dietary energy (Figure 4). In many countries, the HHI-PD score was high, but the percent population with inadequate dietary energy was low.

**Figure 5:**
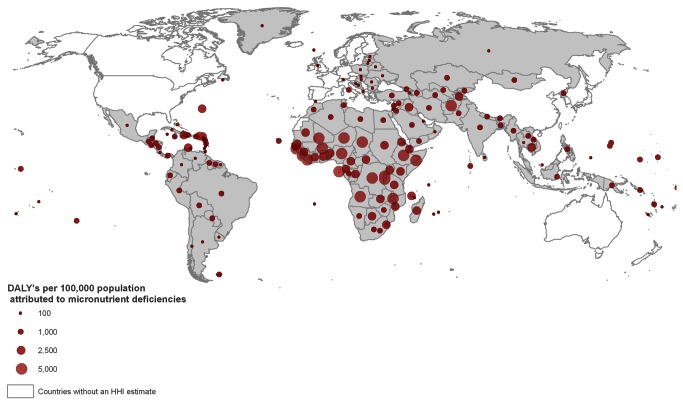
Global map presenting the population-adjusted hidden hunger associated Disability Adjusted Life Years (DALY) (HHI-DBa) in 136 countries. The hidden hunger index HHI-DBa was estimated based on estimates of the DALYs per 100,000 population, attributable to iron, vitamin A, and zinc deficiencies.

**Figure 6:**
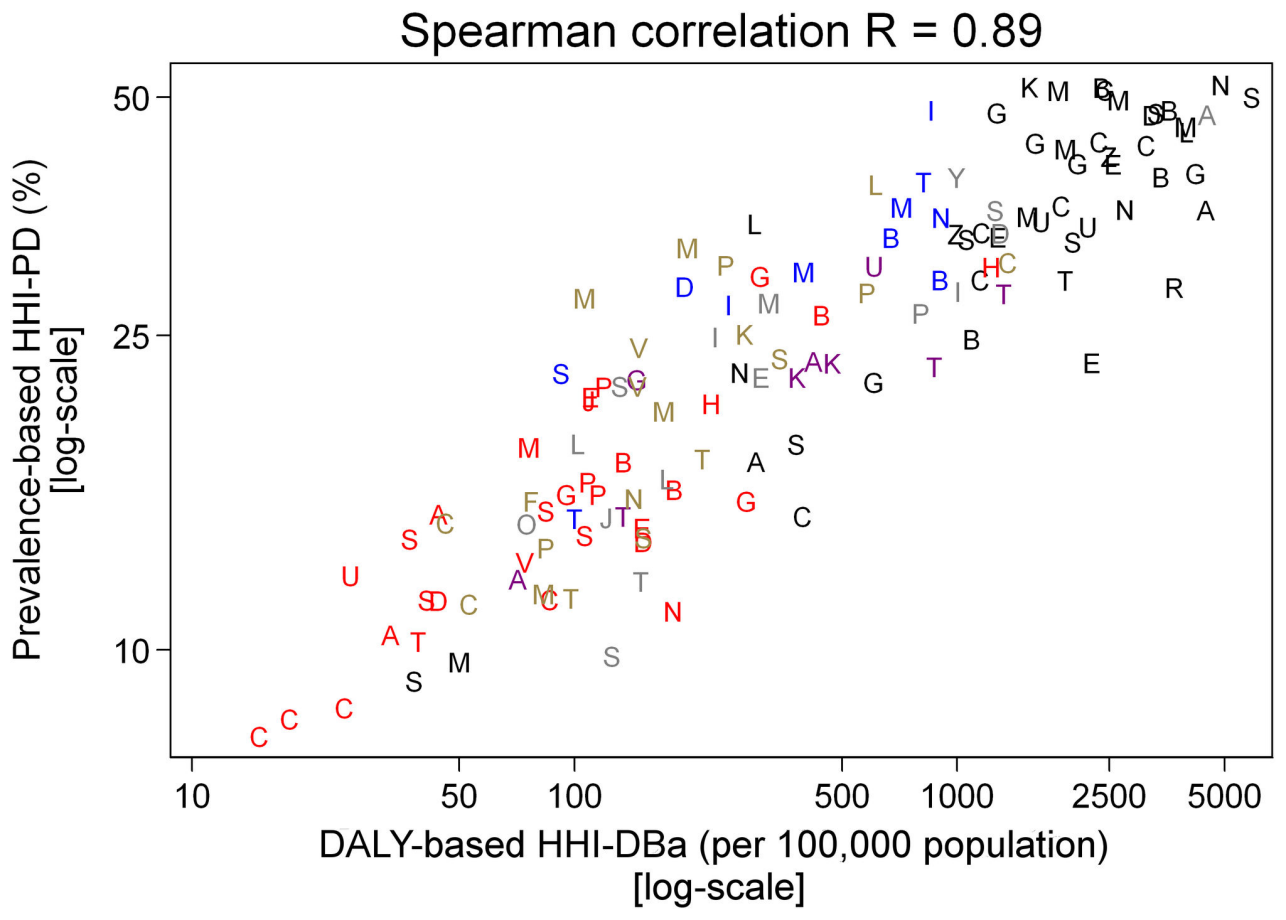
The association between hidden hunger index based on the prevalence estimates (HHI-PD) and hidden hunger associated Disability Adjusted Life Years (DALY) (HHI-DBa) in 133 countries. Prevalence-based HHI-PD estimates were not available for three of the 136 countries with HHI-DBa estimates (the Bahamas, Bahrain, and Somalia).

The DALY-based hidden hunger indices (HHI-DBa and HHI-DBu) were also only calculated for the 136 countries with a 2007 HDI value <0.9 and available DALY estimates (Figure **5** Appendix **S3**). The majority of the countries with high HHI-DBa scores were in sub-Saharan Africa (Figure 5). The disease burden was highest in Sierra Leone, with an estimated total of 5,870 DALYs per 100,000 population, and lowest in Cuba, with 15 DALYs per 100,000 population. Of the top 20 countries, 18 were in sub-Saharan Africa while two, Afghanistan and India, were in Asia. Thirteen of the 20 countries with the highest DALY rates (HHI-DBa) were also among the countries with the highest HHI-PD scores. A Spearman rank correlation of 0.89 was observed between HHI-PD and HHI-DBa (Figure 6).

**Figure 7:**
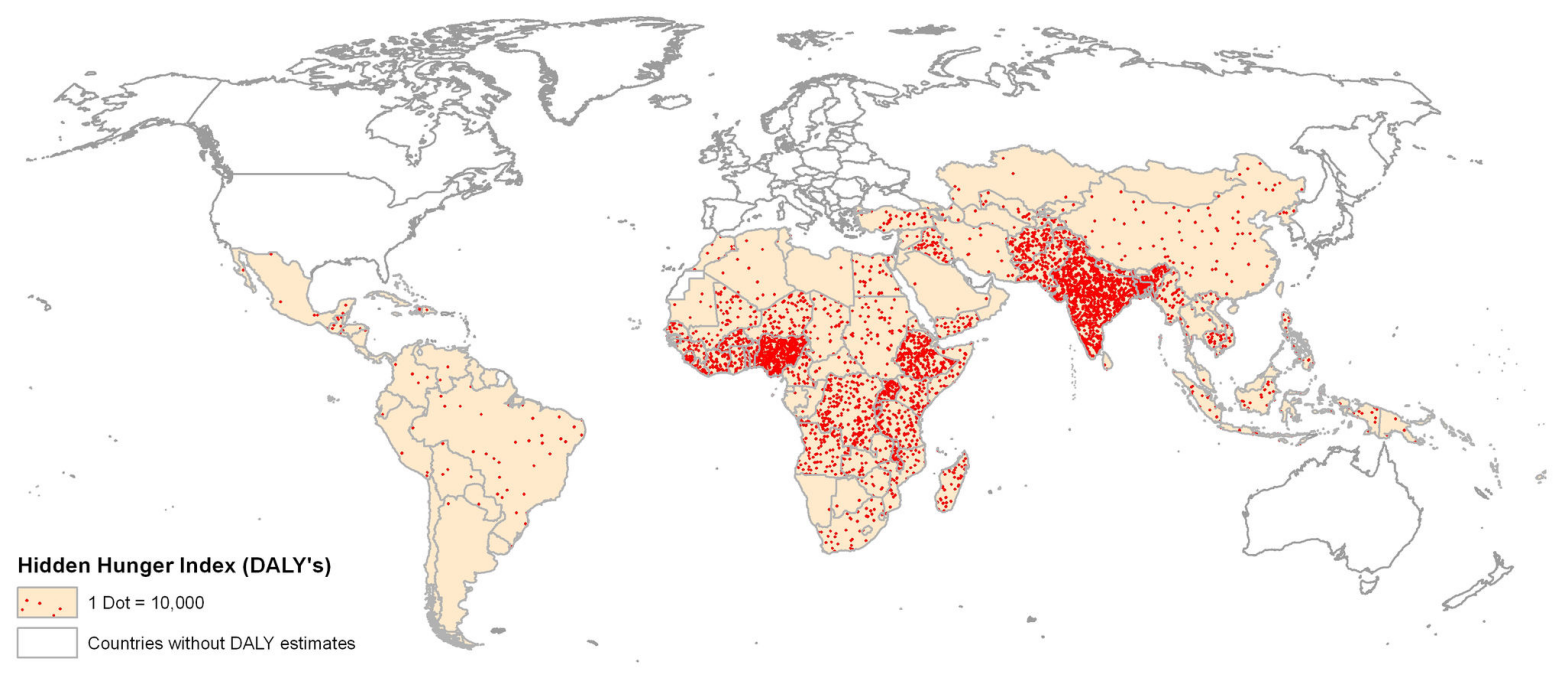
Total population-unadjusted Disability Adjusted Life Years (DALYs) attributed to micronutrient deficiencies in 136 countries. This includes iron, vitamin A, and zinc micronutrient deficiencies.

Among the top 36 countries with 20% or greater prevalence of childhood stunting, and home to 90% of all stunted children globally, the percent total DALYs attributed to micronutrient deficiencies ranged from 1.5% in South Africa to 12.3% in Côte d’Ivoire, with deficiencies of vitamin A and zinc accountable for the largest proportion (data not shown). Conversely, the HHI-DBu scores were high in South Asian countries, such as India, Bangladesh, and Pakistan (Figure 7).

## Discussion

The maps and indices described in this paper provide much-needed information on the collective magnitude and distribution of multiple micronutrient deficiencies across the globe, and their attributed disease burden, for potential use in advocacy and planning efforts to guide health and nutrition policies. Notably, a number of countries in sub-Saharan Africa, as well as India and Afghanistan, had an alarmingly high level of hidden hunger, with stunting, IDA, and VAD all being highly prevalent amongst preschool-age children. Countries in sub-Saharan Africa, such as Sierra Leone and Niger, exhibited the highest levels of population-adjusted disease burden attributed to micronutrient deficiencies. In the 36 high-burden countries, deficiencies of micronutrients, especially vitamin A and zinc, were responsible for 2-12% of the total DALYs. In contrast, due to the large population size of South Asia, the population-unadjusted total DALYs attributed to hidden hunger were greatest in India, Bangladesh, and Pakistan.

The global hidden hunger indices and maps capture the collective burden of micronutrient deficiencies and their contribution to the disease burden. Earlier indices such as the Global Hunger Index (GHI), reflecting measures of food security, undernutrition, and child mortality, capture the multidimensional aspects and consequences of hunger, caused mainly by food and caloric deficit, and do not take into account the burden and consequences of pervasive hidden hunger [31,32]. Maps depicting single micronutrient deficiencies have served to inform policy makers and the scientific community of the extent of individual vitamin and mineral deficiencies, but do not illustrate the more commonly observed multiple micronutrient deficiencies. This offers the public health community and policy makers a novel opportunity to develop a unified and comprehensive approach to targeting the alleviation of multiple micronutrient deficiencies in high burden countries, which is essential to achieving most of the MDGs, and is at the core of the Scaling Up Nutrition (SUN) Movement’s Road Map, which focuses on implementing evidence-based nutrition interventions and integrating nutrition goals across sectors, including health, social protection, poverty alleviation, national development, and agriculture [33].

Deficiencies of various micronutrients often share a common etiology (for example, low consumption of food from animal, fruit, and vegetable origin, or losses due to frequent infections), thereby tending to co-occur and correlate in the same population. In this analysis, there were moderate to high correlations between the prevalence of stunting, anemia, and low serum retinol among preschool children at a country level (data not shown). Similar trends were observed when the prevalence estimates were only based on national survey data, excluding regression-based estimates (data not shown). This suggests that the associations were robust, and not an artifact of the regression models used by the WHO to estimate the prevalence of VAD and anemia for countries with missing national survey data.

Iodine deficiency estimates were an exception, in that they did not correlate with the prevalence of stunting, anemia, and VAD. Consequently, the HHI-PD tabulated for the 149 countries represented the combined prevalence of stunting, anemia due to iron deficiency, and VAD. Estimates of ID were not incorporated but presented separately, due to the lack of correlation with deficiencies of other micronutrients. The greatest proportions of children with ID were in the Eastern Mediterranean (46.6%), European (44.2%), and African (40.4%) regions. However, the high prevalence of ID in populations must be viewed with caution, due to the high intra-individual variability in urinary iodine in both spot and 24-hour urine collections in populations with adequate iodine intake. The varying coverage of salt iodization, combined with the fact that sources of iodine are different from other micronutrients, sets it apart from other deficiencies that cluster together. However, there could be exceptions among these countries, particularly with regard to ID, where salt iodization is no longer mandatory, and the prevalence of low UI concentration is high. Further, other micronutrient deficiencies, such as folate and vitamin B12, not addressed in this paper may be prevalent in these countries.

Countries in sub-Saharan Africa, the only developing region where the numbers of malnourished children have been rising in recent years, exhibited the highest rates of hidden hunger [34]. Low quality diets, as well as frequent infections, are likely to be the key causal factors, further compounded by poor economic conditions and repressive political systems. Asia, India, and Afghanistan exhibited the most severe magnitude of multiple micronutrient deficiencies. India is host to the largest number of undernourished children in the world [35]. It is widely believed that India’s limited success in dealing with undernutrition is linked to poor governance, including the lack of a strong national agenda against malnutrition within the highest executive offices; a lack of consistent monitoring of the situation based on reliable data; and an inability to comprehend malnutrition as a holistic issue, which is affected by the quality of interventions across a number of sectors, including water and sanitation, education, agriculture, and others. Instead, malnutrition is viewed primarily as a problem of hunger and food distribution, which can be dealt with through supplementary feeding and subsidized distribution systems [36].

There was a strong inverse correlation between HHI-PD and HDI, regardless of the use of only national estimates or both national and regression estimates in the calculation of HHI-PD (data not shown). As expected, countries with high HDI tended to have low HHI-PD, and vice versa. This highlights the importance of addressing hidden hunger in order to reduce general deprivation, improve health and education, and vice versa. Conversely, the HHI-PD was only weakly associated with the measure of undernourishment which reflects the proportion of populations with an inadequate energy intake. This indicates that the HHI-PD measures a form of hunger associated less with energy deficiency, and more with a lack of essential micronutrients. The current indices and maps are therefore particularly helpful while planning program assistance for populations which suffer from deficiencies of one or more micronutrients.

The DALY-based indices and maps were intended to capture the consequences of micronutrient deficiencies globally. The population-adjusted DALY rates attributed to micronutrient deficiencies were largest in sub-Saharan African countries, as was observed in hidden hunger prevalence estimates. The DALYs attributed to deficiencies of iron, vitamin A, and zinc were strongly correlated with one another (data not shown). Overall, the hidden hunger indices based on prevalence estimates and DALYs were strongly correlated (r=0.9), implying, as expected, that the disease burden due to hidden hunger tended to be greater in countries where micronutrient deficiencies were prevalent. By contrast, the population-unadjusted total disease burdens attributable to hidden hunger were greatest in countries with large populations in South Asia.

A few limitations of the indices and maps need to be considered. The indices were not comprehensive in their representation of global hidden hunger due to the limited availability of national data pertaining to key micronutrients. In the absence of biochemical indicators of zinc deficiency and the validation of the adequacy of zinc in national food supplies, national stunting prevalence was used to reflect population zinc status, recognizing that this does not reflect the true prevalence of zinc deficiency, and that multiple factors besides zinc deficiency could lead to impaired linear growth. Moreover, to arrive at national estimates of IDA, an assumption of 50-60% of anemia attributable to iron deficiency was made. These assumptions need further validation, however. The HHI-PD estimates were based on prevalence data for preschool-age children, without taking into account other important and recognized vulnerable groups, such as pregnant women. Overall, due to poor data coverage of micronutrient deficiency variables, the HHI-PD could only be estimated for approximately 60% of the countries. The prevalence estimates derived from regression methods for countries lacking national data were, at best, an approximation, which may not accurately reflect the true burden of hidden hunger. In addition, estimates of the correlation between HHI-PD and HHI-DBa must be considered with respect to the fact that there is an overlap in the data on anemia, stunting, and serum retinol used to calculate both indices.

In conclusion, more high-quality national data on the deficiency status of other key vitamins and minerals and a better estimation of zinc and iron deficiency are warranted to improve the measure of global hidden hunger. The regression estimates for anemia and VAD may need to be updated, in order to take into account more recent country prevalence of deficiencies and covariates measuring health status and development indicators. Despite these further needs, the current indices and maps capturing the burden and consequences of hidden hunger provide crucial evidence for the appropriate targeting and prioritizing of comprehensive and inclusive program assistance aiming to tackle global multiple micronutrient deficiencies. Moreover, the indices and maps are believed to serve as useful tools to call for urgent and unified efforts to stimulate relevant global advocacy efforts towards the continued scaling up of nutrition interventions.

### Global hidden hunger indices and maps as an advocacy tool

The current and growing support for the Scaling up Nutrition (SUN) Movement illustrates the unprecedented global political will to prioritize food and nutrition security as being central to the development and achievement of the MDGs. The main investors in SUN are national governments themselves. Governments require tools which enable them to make informed policy and budget decisions. The current hidden hunger indices and maps provide advocates with a tool to further empower decision makers to better understand and visualize the importance of prioritizing interventions that address hidden hunger. This is critical, if political will is to be transformed into effective and scaled-up nutrition direct and nutrition-sensitive interventions.

## Conclusion

Globally, an estimated two billion people are affected by deficiencies of essential vitamins and minerals, collectively known as hidden hunger, which negatively impact on health and economic development. The hidden hunger indices and maps illustrate both the burden of multiple micronutrient deficiencies and their contribution to the disease burden. They also provide a useful tool for advocates to illustrate the real need for multiple micronutrient interventions to address hidden hunger. In addition, they provide useful information for policy makers in decision making and prioritizing interventions, and offer valuable information for public health scientists as a basis for action, and the subsequent monitoring and evaluation of preventive programs.

Of the 20 countries with the highest HHI-PD scores, 18 were in sub-Saharan Africa and two were in Asia. Stunting, iron deficiency anemia, and vitamin A deficiency were highly prevalent amongst preschool children in countries with the highest HHI-PD. The hidden hunger indices provide evidence for the appropriate targeting and prioritizing of comprehensive and inclusive nutrition programs which address global hidden hunger. The HHI-PD and maps also provide valuable information on hot spots where the prevalence of hidden hunger is alarmingly high, and where focused and scaled-up interventions are most critical to the attainment of the MDGs.

## Supporting Information

Appendix S1 Hidden Hunger Index (HHI) scores by country and region.

Appendix S2 Prevalence of micronutrient deficiencies among preschool-aged children and school-aged children (**for low urinary iodine**) in 41 countries with a 2007 Human Development Index (**HDI**) value >0.9 and excluded from estimation of the hidden hunger indices.

Appendix S3 Population adjusted DALY estimates by country and region.
