# Supplementary material for: The Global Hidden Hunger Indices and Maps: An Advocacy Tool for Action
Source: PLoS One. 2013 Jun 12;8(6):e67860. doi: 10.1371/journal.pone.0067860 (PMC3680387; doi:10.1371/journal.pone.0067860)
Supplement: Appendix S1 [file pone.0067860.s001.docx]

**Appendix S1. Hidden Hunger Index (HHI) scores by country and region**

| **Rank** | **Country Name** | **WHO Region** | **HHI** | **Prevalence (%)** | | |
| --- | --- | --- | --- | --- | --- | --- |
|  |  |  |  | **Stunting** | **Anemia due to iron deficiency** | **Low serum retinol**  **(<0.7 μmol/L)** |
| 1 | Niger | Africa | 52.0 | 47.0 | 41.8 | 67.0 |
| 2 | Kenya | Africa | 51.7 | 35.8 | 34.5 | 84.4 |
| 3 | Benin | Africa | 51.3 | 44.7 | 39.1 | 70.7 |
| 4 | Central African Republic | Africa | 51.0 | 43.0 | 42.1 | 68.2 |
| 5 | Mozambique | Africa | 51.0 | 47.0 | 37.4 | 68.8 |
| 6 | Sierra Leone | Africa | 50.0 | 37.4 | 37.9 | 74.8 |
| 7 | Malawi | Africa | 49.7 | 53.2 | 36.6 | 59.2 |
| 8 | India | South-East Asia | 48.3 | 47.9 | 34.7 | 62.0 |
| 9 | Burkina Faso | Africa | 48.3 | 44.5 | 45.8 | 54.3 |
| 10 | Ghana | Africa | 47.7 | 28.6 | 39.0 | 75.8 |
| 11 | São Tomé and Príncipe | Africa | 47.7 | 29.3 | 18.4 | 95.6 |
| 12 | Afghanistan | Eastern Mediterranean | 47.7 | 59.3 | 19.0 | 64.5 |
| 13 | Democratic Republic of the Congo | Africa | 47.7 | 45.8 | 35.7 | 61.1 |
| 14 | Mali | Africa | 46.0 | 38.5 | 40.7 | 58.6 |
| 15 | Liberia | Africa | 45.3 | 39.4 | 43.4 | 52.9 |
| 16 | Côte d'Ivoire | Africa | 44.0 | 40.1 | 34.5 | 57.3 |
| 17 | Gambia | Africa | 43.7 | 27.6 | 39.7 | 64.0 |
| 18 | Chad | Africa | 43.3 | 44.8 | 35.6 | 50.1 |
| 19 | Madagascar | Africa | 43.0 | 52.8 | 34.2 | 42.1 |
| 20 | Zambia | Africa | 42.0 | 45.8 | 26.5 | 54.1 |
| 21 | Guinea | Africa | 41.3 | 40.0 | 38.0 | 45.8 |
| 22 | Ethiopia | Africa | 41.3 | 50.7 | 26.8 | 46.1 |
| 23 | Guinea-Bissau | Africa | 40.0 | 28.1 | 37.5 | 54.7 |
| 24 | Burundi | Africa | 39.7 | 63.1 | 28.0 | 27.9 |
| 25 | Yemen | Eastern Mediterranean | 39.7 | 57.7 | 34.2 | 27.0 |
| 26 | Timor-Leste | South-East Asia | 39.0 | 55.7 | 15.8 | 45.8 |
| 27 | Lao People's Democratic Republic | Western Pacific | 38.7 | 47.6 | 24.1 | 44.7 |
| 28 | Cameroon | Africa | 36.3 | 36.4 | 34.2 | 38.8 |
| 29 | Myanmar | South-East Asia | 36.3 | 40.6 | 31.6 | 36.7 |
| 30 | Nigeria | Africa | 36.0 | 41.0 | 37.8 | 29.5 |
| 31 | Angola | Africa | 36.0 | 29.0 | 14.9 | 64.3 |
| 32 | Sudan | Eastern Mediterranean | 36.0 | 37.9 | 42.3 | 27.8 |
| 33 | Mauritania | Africa | 35.3 | 24.2 | 34.1 | 47.7 |
| 34 | Nepal | South-East Asia | 35.3 | 49.3 | 24.2 | 32.3 |
| 35 | United Republic of Tanzania | Africa | 35.0 | 44.4 | 35.9 | 24.2 |
| 36 | Lesotho | Africa | 34.7 | 42.0 | 29.2 | 32.7 |
| 37 | Uganda | Africa | 34.3 | 38.7 | 36.3 | 27.9 |
| 38 | Comoros | Africa | 33.7 | 47.0 | 32.7 | 21.5 |
| 39 | Djibouti | Eastern Mediterranean | 33.7 | 33.0 | 32.9 | 35.2 |
| 40 | Zimbabwe | Africa | 33.7 | 35.8 | 29.2 | 35.8 |
| 41 | Eritrea | Africa | 33.3 | 43.7 | 34.8 | 21.4 |
| 42 | Bhutan | South-East Asia | 33.3 | 37.5 | 40.3 | 22.0 |
| 43 | Swaziland | Africa | 33.0 | 29.5 | 25.1 | 44.6 |
| 44 | Senegal | Africa | 32.7 | 20.1 | 41.3 | 37.0 |
| 45 | Marshall Islands | Western Pacific | 32.3 | 18.2 | 18.0 | 60.7 |
| 46 | Cambodia | Western Pacific | 31.0 | 39.5 | 31.0 | 22.3 |
| 47 | Philippines | Western Pacific | 30.7 | 33.8 | 18.2 | 40.1 |
| 48 | Uzbekistan | Europe | 30.7 | 19.6 | 19.1 | 53.1 |
| 49 | Haiti | Americas | 30.7 | 29.0 | 30.6 | 32.0 |
| 50 | Maldives | South-East Asia | 30.0 | 31.9 | 48.9 | 9.4 |
| 51 | Guatemala | Americas | 29.7 | 54.3 | 19.1 | 15.8 |
| 52 | Bangladesh | South-East Asia | 29.3 | 43.0 | 23.5 | 21.7 |
| 53 | Congo | Africa | 29.3 | 31.2 | 32.4 | 24.6 |
| 54 | Togo | Africa | 29.3 | 26.9 | 26.2 | 35.0 |
| 55 | Dem. People's Republic of Korea | South-East Asia | 28.7 | 43.1 | 15.9 | 27.5 |
| 56 | Rwanda | Africa | 28.7 | 51.7 | 28.2 | 6.4 |
| 57 | Iraq | Eastern Mediterranean | 28.3 | 27.5 | 28.0 | 29.8 |
| 58 | Papua New Guinea | Western Pacific | 28.3 | 43.9 | 29.9 | 11.1 |
| 59 | Tajikistan | Europe | 28.3 | 39.0 | 18.9 | 26.8 |
| 60 | Micronesia (Fed. States of) | Western Pacific | 28.0 | 18.2 | 11.2 | 54.2 |
| 61 | Morocco | Eastern Mediterranean | 27.3 | 23.1 | 18.9 | 40.4 |
| 62 | Indonesia | South-East Asia | 27.3 | 40.1 | 22.3 | 19.6 |
| 63 | Pakistan | Eastern Mediterranean | 26.7 | 42.0 | 25.5 | 12.5 |
| 64 | Bolivia | Americas | 26.7 | 27.1 | 30.7 | 21.8 |
| 65 | Kiribati | Western Pacific | 25.0 | 28.3 | 25.1 | 21.8 |
| 66 | Iran (Islamic Republic of) | Eastern Mediterranean | 25.0 | 56.7 | 17.5 | 0.5 |
| 67 | Botswana | Africa | 24.7 | 29.1 | 19.0 | 26.1 |
| 68 | Viet Nam | Western Pacific | 24.0 | 43.3 | 17.1 | 12.0 |
| 69 | Solomon Islands | Western Pacific | 23.3 | 32.8 | 24.2 | 13.1 |
| 70 | Azerbaijan | Europe | 23.3 | 26.8 | 10.7 | 32.1 |
| 71 | Equatorial Guinea | Africa | 23.0 | 35.0 | 20.4 | 13.9 |
| 72 | Kyrgyzstan | Europe | 23.0 | 18.0 | 24.9 | 26.3 |
| 73 | Turkmenistan | Europe | 22.7 | 19.0 | 21.5 | 28.0 |
| 74 | Namibia | Africa | 22.3 | 29.6 | 20.3 | 17.5 |
| 75 | Sri Lanka | South-East Asia | 22.3 | 19.2 | 12.6 | 35.3 |
| 76 | Egypt | Eastern Mediterranean | 22.0 | 30.7 | 23.8 | 11.9 |
| 77 | Kazakhstan | Europe | 22.0 | 17.5 | 21.8 | 27.1 |
| 78 | Georgia | Europe | 22.0 | 14.7 | 20.3 | 30.9 |
| 79 | Gabon | Africa | 21.7 | 26.3 | 22.3 | 16.9 |
| 80 | Syrian Arab Republic | Eastern Mediterranean | 21.7 | 28.0 | 24.6 | 12.1 |
| 81 | Vanuatu | Western Pacific | 21.7 | 19.1 | 29.5 | 16.1 |
| 82 | Peru | Americas | 21.7 | 30.0 | 19.7 | 14.9 |
| 83 | Ecuador | Americas | 21.0 | 29.0 | 19.0 | 14.7 |
| 84 | Jamaica | Americas | 20.7 | 3.7 | 28.9 | 29.4 |
| 85 | Honduras | Americas | 20.3 | 29.0 | 18.7 | 13.8 |
| 86 | Mongolia | Western Pacific | 20.0 | 27.5 | 12.8 | 19.8 |
| 87 | Ukraine | Europe | 20.0 | 22.9 | 13.3 | 23.8 |
| 88 | TFYR Macedonia | Europe | 19.0 | 11.5 | 15.5 | 29.7 |
| 89 | Republic of Moldova | Europe | 18.3 | 10.0 | 19.3 | 25.6 |
| 90 | Lebanon | Eastern Mediterranean | 18.3 | 26.7 | 17.0 | 11.0 |
| 91 | South Africa | Africa | 18.3 | 27.0 | 10.7 | 16.9 |
| 92 | Mexico | Americas | 18.0 | 15.5 | 11.9 | 26.8 |
| 93 | Romania | Europe | 17.7 | 12.8 | 23.9 | 16.3 |
| 94 | Tuvalu | Western Pacific | 17.3 | 10.0 | 20.5 | 21.8 |
| 95 | Algeria | Africa | 17.3 | 15.0 | 21.3 | 15.7 |
| 96 | Belize | Americas | 17.3 | 22.2 | 18.0 | 11.7 |
| 97 | Libyan Arab Jamahiriya | Eastern Mediterranean | 16.3 | 21.0 | 20.3 | 8.0 |
| 98 | Panama | Americas | 16.3 | 21.5 | 18.0 | 9.4 |
| 99 | Albania | Europe | 16.0 | 19.4 | 10.4 | 18.6 |
| 100 | Brazil | Americas | 16.0 | 7.1 | 27.5 | 13.3 |
| 101 | Paraguay | Americas | 15.7 | 18.0 | 15.1 | 14.1 |
| 102 | Grenada | Americas | 15.7 | 13.9 | 19.2 | 14.1 |
| 103 | Niue | Western Pacific | 15.7 | 18.1 | 13.0 | 15.5 |
| 104 | Bulgaria | Europe | 15.3 | 8.8 | 19.4 | 18.3 |
| 105 | Guyana | Americas | 15.3 | 18.2 | 24.0 | 4.1 |
| 106 | Fiji | Western Pacific | 15.3 | 2.7 | 29.9 | 13.6 |
| 107 | Saint Vincent and the Grenadines | Americas | 15.0 | 23.5 | 19.4 | 2.1 |
| 108 | Antigua and Barbuda | Americas | 15.0 | 7.5 | 29.6 | 7.4 |
| 109 | Latvia | Europe | 14.7 | 15.3 | 16.0 | 13.0 |
| 110 | Turkey | Europe | 14.7 | 15.6 | 16.3 | 12.4 |
| 111 | Cape Verde | Africa | 14.7 | 16.2 | 26.1 | 2.0 |
| 112 | Jordan | Eastern Mediterranean | 14.7 | 12.0 | 17.0 | 15.1 |
| 113 | Thailand | South-East Asia | 14.7 | 15.7 | 12.6 | 15.7 |
| 114 | Cook Islands | Western Pacific | 14.3 | 18.2 | 14.8 | 10.4 |
| 115 | Oman | Eastern Mediterranean | 14.3 | 12.9 | 24.9 | 5.5 |
| 116 | Russian Federation | Europe | 14.3 | 13.0 | 15.9 | 14.1 |
| 117 | El Salvador | Americas | 14.3 | 19.0 | 9.2 | 14.6 |
| 118 | Suriname | Americas | 14.0 | 11.0 | 12.9 | 18.0 |
| 119 | Samoa | Western Pacific | 14.0 | 4.2 | 21.3 | 16.1 |
| 120 | Saint Lucia | Americas | 13.7 | 10.8 | 19.3 | 11.3 |
| 121 | Dominican Republic | Americas | 13.7 | 10.1 | 17.3 | 13.7 |
| 122 | Bosnia and Herzegovina | Europe | 13.7 | 11.8 | 16.1 | 13.2 |
| 123 | Lithuania | Europe | 13.7 | 15.3 | 14.3 | 11.1 |
| 124 | Palau | Western Pacific | 13.3 | 18.2 | 13.3 | 8.9 |
| 125 | Venezuela (Bolivarian Republic of) | Americas | 13.0 | 12.8 | 16.6 | 9.4 |
| 126 | Belarus | Europe | 12.7 | 4.5 | 16.4 | 17.4 |
| 127 | Poland | Europe | 12.7 | 15.3 | 13.6 | 9.3 |
| 128 | Estonia | Europe | 12.7 | 15.3 | 14.0 | 8.7 |
| 129 | Slovakia | Europe | 12.7 | 15.3 | 14.0 | 8.3 |
| 130 | Uruguay | Americas | 12.3 | 13.9 | 11.5 | 11.9 |
| 131 | Armenia | Europe | 12.3 | 18.0 | 18.3 | 0.6 |
| 132 | Tunisia | Eastern Mediterranean | 12.3 | 9.0 | 13.0 | 14.6 |
| 133 | Malaysia | Western Pacific | 11.7 | 15.6 | 16.2 | 3.5 |
| 134 | Tonga | Western Pacific | 11.7 | 1.3 | 16.6 | 17.0 |
| 135 | Colombia | Americas | 11.7 | 15.0 | 13.9 | 5.9 |
| 136 | Saint Kitts and Nevis | Americas | 11.7 | 13.9 | 13.7 | 7.1 |
| 137 | Dominica | Americas | 11.7 | 9.8 | 20.6 | 4.2 |
| 138 | China | Western Pacific | 11.3 | 15.0 | 10.0 | 9.3 |
| 139 | Nicaragua | Americas | 11.3 | 22.0 | 8.5 | 3.1 |
| 140 | Argentina | Americas | 10.3 | 8.0 | 9.1 | 14.3 |
| 141 | Trinidad and Tobago | Americas | 10.3 | 5.3 | 18.2 | 7.2 |
| 142 | Saudi Arabia | Eastern Mediterranean | 9.7 | 9.3 | 16.6 | 3.6 |
| 143 | Mauritius | Africa | 9.7 | 9.7 | 10.1 | 9.2 |
| 144 | Seychelles | Africa | 9.0 | 5.1 | 14.3 | 8.0 |
| 145 | Costa Rica | Americas | 8.3 | 6.1 | 10.5 | 8.8 |
| 146 | Chile | Americas | 8.3 | 2.0 | 14.6 | 7.9 |
| 147 | Croatia | Europe | 8.0 | 1.0 | 14.0 | 9.2 |
| 148 | Cuba | Americas | 7.7 | 3.7 | 16.0 | 3.6 |
| 149 | Hungary | Europe | 7.3 | 3.3 | 11.3 | 7.0 |
