# Supplementary material for: The Global Hidden Hunger Indices and Maps: An Advocacy Tool for Action
Source: PLoS One. 2013 Jun 12;8(6):e67860. doi: 10.1371/journal.pone.0067860 (PMC3680387; doi:10.1371/journal.pone.0067860)
Supplement: Appendix S2 [file pone.0067860.s002.docx]

**Appendix S2. Prevalence of micronutrient deficiencies among preschool-aged children and school-aged children (for low urinary iodine) in 41 countries with a 2007 Human Development Index (HDI) value >0.9 and excluded from estimating of the hidden hunger indices**

| **WHO Region** | **Country** | **2007 HDI^1^** | **Prevalence estimates (%)** | | |
| --- | --- | --- | --- | --- | --- |
|  |  |  | **Stunting** | **Anemia due to iron deficiency** | **Low serum retinol**  **(<0.7 μmol/L)** |
| Europe | Andorra | 0.934 |  | 7.2 |  |
|  | Austria | 0.955 |  | 6.3 |  |
|  | Belgium | 0.953 |  | 5.2 |  |
|  | Cyprus | 0.914 |  | 11.2 |  |
|  | Czech Republic | 0.955 | 2.6 | 11.0 | 5.8 |
|  | Denmark | 0.955 |  | 5.4 |  |
|  | Finland | 0.959 |  | 6.9 |  |
|  | France | 0.961 |  | 5.0 |  |
|  | Germany | 0.947 | 1.3 | 4.7 |  |
|  | Greece | 0.942 |  | 7.3 |  |
|  | Iceland | 0.969 |  | 4.7 |  |
|  | Ireland | 0.965 |  | 6.2 |  |
|  | Israel | 0.935 |  | 7.1 |  |
|  | Italy | 0.951 |  | 6.5 |  |
|  | Liechtenstein | 0.951 |  |  |  |
|  | Luxembourg | 0.96 |  | 5.6 |  |
|  | Malta | 0.902 |  | 9.8 | 4.0 |
|  | Monaco | 0.948 |  | 3.0 |  |
|  | Netherlands | 0.964 | 1.8 | 5.2 |  |
|  | Norway | 0.971 |  | 3.8 |  |
|  | Portugal | 0.909 |  | 7.6 |  |
|  | San Marino | 0.94 |  | 5.5 |  |
|  | Slovenia | 0.929 |  | 8.4 |  |
|  | Spain | 0.955 |  | 7.7 |  |
|  | Sweden | 0.963 |  | 5.2 |  |
|  | Switzerland | 0.96 |  | 3.8 |  |
|  | United Kingdom | 0.947 |  | 4.8 |  |
| Americas | Barbados | 0.903 | 10.2 | 10.3 | 6.5 |
|  | Canada | 0.966 |  | 4.6 |  |
|  | United States of America | 0.956 | 3.9 | 1.9 |  |
| Western Pacific | Australia | 0.97 |  | 4.8 |  |
|  | Brunei Darussalam | 0.92 | 11.6 | 14.5 |  |
|  | Hong Kong | 0.944 | 18.2 |  |  |
|  | Japan | 0.96 | 5.6 | 6.4 |  |
|  | New Zealand | 0.948 | 2.9 | 6.8 |  |
|  | Republic of Korea | 0.937 | 18.3 | 8.3 |  |
|  | Singapore | 0.944 | 4.4 | 11.3 |  |
| Pacific Island | Nauru | 0.906 | 24.0 | 14.4 | 10.0 |
| Eastern Mediterranean | Kuwait | 0.916 | 24.0 | 14.0 |  |
|  | Qatar | 0.91 | 8.1 | 15.7 |  |
|  | United Arab Emirates | 0.903 | 17.0 | 16.6 |  |

^1^ For countries with no HDI values (n=41), 2007 life expectancy index values, one of the main components of HDI, was used.
