# Supplementary material for: The Global Hidden Hunger Indices and Maps: An Advocacy Tool for Action
Source: PLoS One. 2013 Jun 12;8(6):e67860. doi: 10.1371/journal.pone.0067860 (PMC3680387; doi:10.1371/journal.pone.0067860)
Supplement: Appendix S3 [file pone.0067860.s003.docx]

**Appendix S3. Population adjusted DALY estimates by country and region**

| **Rank** | **Country Name** | **WHO Region** | **HHI DALYs per 100,000 population** | **DALYs per 100,000 population** | | |
| --- | --- | --- | --- | --- | --- | --- |
|  |  |  |  | **Iron deficiency** | **Vitamin A deficiency** | **Zinc deficiency** |
| 1 | Sierra Leone | Africa | 5870 | 461 | 2835 | 2574 |
| 2 | Niger | Africa | 4880 | 159 | 2193 | 2528 |
| 3 | Afghanistan | Eastern Mediterranean | 4491 | 182 | 2317 | 1993 |
| 4 | Angola | Africa | 4459 | 75 | 2125 | 2258 |
| 5 | Somalia | Eastern Mediterranean | 4198 | 350 | 2511 | 1337 |
| 6 | Guinea-Bissau | Africa | 4188 | 325 | 2094 | 1768 |
| 7 | Liberia | Africa | 3969 | 278 | 2252 | 1439 |
| 8 | Mali | Africa | 3952 | 116 | 1969 | 1867 |
| 9 | Rwanda | Africa | 3701 | 181 | 2016 | 1503 |
| 10 | Burkina Faso | Africa | 3578 | 360 | 1394 | 1824 |
| 11 | Burundi | Africa | 3401 | 148 | 1709 | 1544 |
| 12 | São Tomé and Príncipe | Africa | 3306 | 1909 | 991 | 407 |
| 13 | Democratic Republic of the Congo | Africa | 3224 | 65 | 1505 | 1654 |
| 14 | Chad | Africa | 3126 | 93 | 1587 | 1446 |
| 15 | Nigeria | Africa | 2745 | 53 | 1306 | 1386 |
| 16 | Malawi | Africa | 2635 | 184 | 1299 | 1152 |
| 17 | Ethiopia | Africa | 2552 | 168 | 1372 | 1012 |
| 18 | Zambia | Africa | 2493 | 105 | 1168 | 1220 |
| 19 | Central African Republic | Africa | 2433 | 59 | 1276 | 1099 |
| 20 | Benin | Africa | 2382 | 139 | 1007 | 1236 |
| 21 | Côte d'Ivoire | Africa | 2352 | 65 | 1294 | 993 |
| 22 | Equatorial Guinea | Africa | 2245 | 73 | 1043 | 1129 |
| 23 | Uganda | Africa | 2197 | 108 | 1002 | 1087 |
| 24 | Guinea | Africa | 2059 | 82 | 919 | 1058 |
| 25 | Senegal | Africa | 2000 | 51 | 921 | 1028 |
| 26 | Madagascar | Africa | 1920 | 48 | 1123 | 748 |
| 27 | Togo | Africa | 1919 | 41 | 794 | 1084 |
| 28 | Cameroon | Africa | 1873 | 53 | 737 | 1083 |
| 29 | Mozambique | Africa | 1837 | 110 | 948 | 779 |
| 30 | United Republic of Tanzania | Africa | 1658 | 78 | 812 | 767 |
| 31 | Gambia | Africa | 1596 | 108 | 787 | 701 |
| 32 | Kenya | Africa | 1545 | 106 | 786 | 653 |
| 33 | Mauritania | Africa | 1526 | 51 | 790 | 685 |
| 34 | Cambodia | Western Pacific | 1356 | 206 | 621 | 529 |
| 35 | Tajikistan | Europe | 1323 | 63 | 773 | 487 |
| 36 | Djibouti | Eastern Mediterranean | 1298 | 45 | 856 | 397 |
| 37 | Eritrea | Africa | 1277 | 119 | 708 | 450 |
| 38 | Ghana | Africa | 1268 | 44 | 573 | 651 |
| 39 | Sudan | Eastern Mediterranean | 1255 | 94 | 666 | 496 |
| 40 | Haiti | Americas | 1230 | 374 | 330 | 525 |
| 41 | Comoros | Africa | 1160 | 71 | 741 | 349 |
| 42 | Congo | Africa | 1150 | 51 | 495 | 605 |
| 43 | Botswana | Africa | 1088 | 30 | 693 | 365 |
| 44 | Swaziland | Africa | 1055 | 65 | 700 | 289 |
| 45 | Iraq | Eastern Mediterranean | 1006 | 132 | 506 | 367 |
| 46 | Yemen | Eastern Mediterranean | 996 | 76 | 472 | 449 |
| 47 | Zimbabwe | Africa | 991 | 19 | 583 | 389 |
| 48 | Nepal | South-East Asia | 908 | 51 | 577 | 280 |
| 49 | Bangladesh | South-East Asia | 901 | 29 | 606 | 267 |
| 50 | Turkmenistan | Europe | 872 | 29 | 544 | 299 |
| 51 | India | South-East Asia | 855 | 42 | 559 | 255 |
| 52 | Timor-Leste | South-East Asia | 817 | 113 | 362 | 341 |
| 53 | Pakistan | Eastern Mediterranean | 802 | 31 | 574 | 197 |
| 54 | Myanmar | South-East Asia | 716 | 31 | 395 | 289 |
| 55 | Bhutan | South-East Asia | 671 | 39 | 434 | 198 |
| 56 | Lao People's Democratic Republic | Western Pacific | 612 | 44 | 375 | 193 |
| 57 | Uzbekistan | Europe | 607 | 22 | 394 | 190 |
| 58 | Gabon | Africa | 604 | 21 | 281 | 302 |
| 59 | Papua New Guinea | Western Pacific | 580 | 100 | 403 | 77 |
| 60 | Kyrgyzstan | Europe | 471 | 36 | 312 | 123 |
| 61 | Bolivia | Americas | 442 | 38 | 195 | 209 |
| 62 | Azerbaijan | Europe | 421 | 140 | 147 | 135 |
| 63 | Maldives | South-East Asia | 397 | 44 | 240 | 113 |
| 64 | Cape Verde | Africa | 394 | 72 | 252 | 71 |
| 65 | Kazakhstan | Europe | 381 | 17 | 268 | 97 |
| 66 | South Africa | Africa | 379 | 47 | 232 | 100 |
| 67 | Solomon Islands | Western Pacific | 343 | 105 | 193 | 45 |
| 68 | Morocco | Eastern Mediterranean | 322 | 17 | 236 | 68 |
| 69 | Egypt | Eastern Mediterranean | 307 | 17 | 202 | 89 |
| 70 | Guatemala | Americas | 305 | 38 | 112 | 155 |
| 71 | Algeria | Africa | 298 | 21 | 226 | 51 |
| 72 | Lesotho | Africa | 295 | 43 | 224 | 27 |
| 73 | Guyana | Americas | 281 | 62 | 120 | 99 |
| 74 | Kiribati | Western Pacific | 278 | 49 | 202 | 27 |
| 75 | Namibia | Africa | 270 | 52 | 146 | 72 |
| 76 | Indonesia | South-East Asia | 253 | 22 | 162 | 69 |
| 77 | Philippines | Western Pacific | 248 | 26 | 134 | 88 |
| 78 | Iran (Islamic Republic of) | Eastern Mediterranean | 233 | 18 | 161 | 54 |
| 79 | Honduras | Americas | 228 | 38 | 80 | 111 |
| 80 | Tuvalu | Western Pacific | 216 | 88 | 104 | 24 |
| 81 | Marshall Islands | Western Pacific | 197 | 22 | 147 | 28 |
| 82 | Dem. People's Republic of Korea | South-East Asia | 194 | 13 | 53 | 128 |
| 83 | Brazil | Americas | 182 | 41 | 78 | 63 |
| 84 | Nicaragua | Americas | 181 | 23 | 68 | 89 |
| 85 | Libyan Arab Jamahiriya | Eastern Mediterranean | 174 | 19 | 121 | 35 |
| 86 | Mongolia | Western Pacific | 171 | 11 | 54 | 106 |
| 87 | Dominican Republic | Americas | 151 | 20 | 76 | 55 |
| 88 | Samoa | Western Pacific | 151 | 73 | 62 | 15 |
| 89 | El Salvador | Americas | 150 | 40 | 53 | 57 |
| 90 | Tunisia | Eastern Mediterranean | 149 | 10 | 106 | 32 |
| 91 | Viet Nam | Western Pacific | 147 | 17 | 84 | 45 |
| 92 | Vanuatu | Western Pacific | 146 | 46 | 83 | 17 |
| 93 | Georgia | Europe | 145 | 12 | 79 | 53 |
| 94 | Niue | Western Pacific | 143 | 117 | 19 | 8 |
| 95 | Belize | Americas | 134 | 50 | 45 | 39 |
| 96 | Turkey | Europe | 134 | 20 | 68 | 46 |
| 97 | Syrian Arab Republic | Eastern Mediterranean | 131 | 47 | 49 | 34 |
| 98 | Saudi Arabia | Eastern Mediterranean | 125 | 19 | 62 | 45 |
| 99 | Jordan | Eastern Mediterranean | 121 | 16 | 62 | 43 |
| 100 | Peru | Americas | 119 | 26 | 50 | 43 |
| 101 | Paraguay | Americas | 115 | 22 | 53 | 40 |
| 102 | Ecuador | Americas | 110 | 21 | 54 | 34 |
| 103 | Jamaica | Americas | 109 | 28 | 48 | 33 |
| 104 | Panama | Americas | 108 | 19 | 39 | 51 |
| 105 | Micronesia (Fed. States of) | Western Pacific | 106 | 35 | 56 | 16 |
| 106 | Suriname | Americas | 106 | 12 | 53 | 42 |
| 107 | Lebanon | Eastern Mediterranean | 102 | 15 | 55 | 32 |
| 108 | Thailand | South-East Asia | 100 | 9 | 60 | 31 |
| 109 | Tonga | Western Pacific | 98 | 31 | 56 | 11 |
| 110 | Grenada | Americas | 95 | 37 | 33 | 25 |
| 111 | Sri Lanka | South-East Asia | 92 | 12 | 60 | 20 |
| 112 | Colombia | Americas | 86 | 16 | 45 | 25 |
| 113 | Palau | Western Pacific | 84 | 31 | 42 | 11 |
| 114 | Saint Vincent and the Grenadines | Americas | 84 | 18 | 47 | 19 |
| 115 | Malaysia | Western Pacific | 83 | 18 | 46 | 19 |
| 116 | Fiji | Western Pacific | 77 | 40 | 30 | 6 |
| 117 | Mexico | Americas | 76 | 18 | 28 | 30 |
| 118 | Oman | Eastern Mediterranean | 75 | 25 | 29 | 21 |
| 119 | Venezuela (Bolivarian Republic of) | Americas | 74 | 17 | 36 | 21 |
| 120 | Armenia | Europe | 71 | 6 | 42 | 23 |
| 121 | China | Western Pacific | 53 | 7 | 22 | 25 |
| 122 | Mauritius | Africa | 50 | 10 | 32 | 8 |
| 123 | Cook Islands | Western Pacific | 46 | 13 | 26 | 6 |
| 124 | Antigua and Barbuda | Americas | 44 | 23 | 17 | 5 |
| 125 | Dominica | Americas | 44 | 17 | 21 | 5 |
| 126 | Saint Kitts and Nevis | Americas | 41 | 11 | 20 | 10 |
| 127 | Trinidad and Tobago | Americas | 39 | 12 | 20 | 6 |
| 128 | Seychelles | Africa | 38 | 10 | 23 | 5 |
| 129 | Saint Lucia | Americas | 37 | 14 | 18 | 6 |
| 130 | Argentina | Americas | 33 | 8 | 19 | 7 |
| 131 | Bahamas | Americas | 26 | 9 | 10 | 7 |
| 132 | Uruguay | Americas | 26 | 8 | 13 | 6 |
| 133 | Costa Rica | Americas | 25 | 9 | 9 | 7 |
| 134 | Bahrain | Eastern Mediterranean | 20 | 10 | 7 | 3 |
| 135 | Chile | Americas | 18 | 9 | 6 | 3 |
| 136 | Cuba | Americas | 15 | 8 | 4 | 3 |
